# Single cell RNA-sequencing profiling to improve the translation between human IBD and *in vivo* models

**DOI:** 10.3389/fimmu.2023.1291990

**Published:** 2023-12-19

**Authors:** Erik P. Karmele, Ana Laura Moldoveanu, Irem Kaymak, Bat-Erdene Jugder, Rebecca L. Ursin, Kyle J. Bednar, Daniele Corridoni, Tatiana Ort

**Affiliations:** ^1^ Bioscience Immunology, Research and Early Development, Respiratory and Immunology, Biopharmaceuticals R&D, AstraZeneca, Gaithersburg, MD, United States; ^2^ Bioscience Immunology, Research and Early Development, Respiratory and Immunology, Biopharmaceuticals R&D, AstraZeneca, Cambridge, United Kingdom; ^3^ Bioscience Immunology, Research and Early Development, Respiratory and Immunology, Biopharmaceuticals R&D, AstraZeneca, Waltham, MA, United States

**Keywords:** pre-clinical models, Inflammatory bowel disease (IBD), single cell RNA-sequencing (scRNA-seq), epithelial cell, stromal cell, immune cell

## Abstract

Inflammatory bowel disease (IBD) is an umbrella term for two conditions (Crohn’s Disease and Ulcerative Colitis) that is characterized by chronic inflammation of the gastrointestinal tract. The use of pre-clinical animal models has been invaluable for the understanding of potential disease mechanisms. However, despite promising results of numerous therapeutics in mouse colitis models, many of these therapies did not show clinical benefits in patients with IBD. Single cell RNA-sequencing (scRNA-seq) has recently revolutionized our understanding of complex interactions between the immune system, stromal cells, and epithelial cells by mapping novel cell subpopulations and their remodeling during disease. This technology has not been widely applied to pre-clinical models of IBD. ScRNA-seq profiling of murine models may provide an opportunity to increase the translatability into the clinic, and to choose the most appropriate model to test hypotheses and novel therapeutics. In this review, we have summarized some of the key findings at the single cell transcriptomic level in IBD, how specific signatures have been functionally validated *in vivo*, and highlighted the similarities and differences between scRNA-seq findings in human IBD and experimental mouse models. In each section of this review, we highlight the importance of utilizing this technology to find the most suitable or translational models of IBD based on the cellular therapeutic target.

## Introduction

Inflammatory bowel disease (IBD), consisting of ulcerative colitis (UC) and Crohn’s disease (CD), is a chronic relapsing and remitting inflammatory mediated disorder of the lining of the gastrointestinal tract, resulting in clinical symptoms (1). Changes and functional alterations of immune cells and non-hematopoietic cells (including epithelial and stromal cells) are hallmarks of the onset and sustained inflammation associated with IBD (2, 3). Much of what we know about human IBD pathogenesis is attributed to the use of numerous pre-clinical models of IBD. These models include chemically-induced, spontaneous, immune cell-induced, and microbiota-dependent colitis models. These models have been extensively reviewed (4, 5) and the most common pre-clinical IBD models are summarized in **Table 1**. While numerous pre-clinical models of IBD exist, no single model fully recapitulates the complexity observed in human IBD. Thus, there remains a need to further validate these pre-clinical models of IBD and identify mucosal cell subpopulations with their associated disease-relevant phenotype which may overlay with human IBD. This understanding will potentially increase their translatability into the clinic and help define the best model to test novel therapeutic targets and modalities.

**Table 1:** Summary of the most commonly used pre-clinical models of IBD.

Classification	Type	Mechanism	Advantages	References
Chemical	Dextran Sodium Sulfate (DSS)	Destruction of colonocytes and dissemination of microbial and dietary contents into the underlying lamina propria	Can induce both acute and chronic inflammation, Can model “flare” and “remission” periods of human disease	(6)
Chemical	Trinitrobenzene sulfonic acid (TNBS)	TNBS serves as a hapten that couples with intestinal antigens that drive immunogenic responses	Drives robust TH1 and TH17 immune responses in the colon tissue	(7, 8)
Chemical	Oxazolone	Oxazalone serves as a haptenizing agent	Model can be used to interrogate type-2 immune responses, specifically IL-13 and the role of NKT cells	(9, 10)
Immune cell-mediated	T cell adoptive transfer model	Naïve CD4^+^ or CD8^+^ T cells become activated by the microbiota in the absence of Tregs and secrete IFNγ and IL-17A	Can study T cell subsets, polarization, and migration into the lamina propria, Can study the role of Treg suppression of effector CD4^+^ T cells	(11, 12)
Genetically Engineered	*Il10^-/-^*	Develop spontaneous colitis characterized by progressive immune cell infiltration	This model has allowed for a better understanding of the impact of the microbiome on intestinal inflammation	(13, 14)
Antibody-induced	Anti-CD40	Agonistic anti-CD40 mAb activates dendritic cells and monocytes to secrete cytokines including IL-12 and IL-23, driving the secretion of IFNγ from NK cells	This model can be used to study the role of innate immune cells in intestinal inflammation	(15)
Spontaneous	TRUC	Spontaneous colitis develops by the production of TNFα and IL-23 in response to the microbiota due to T-bet deficiencies in the innate immune system	Initial model that identified a colitogenic microbiota. Treatment of TRUC mice with anti-TNF alleviates inflammation	(16, 17)
Spontaneous	SAMP1YitFc	Generated by interbreeding of AKR mice. Spontaneous inflammation develops in the terminal ileum and cecum, and is driven by TH1 immune response.	A pre-clinical model that displays inflammation in the terminal ileum and the administration of anti-TNF alleviates inflammation – relevant for Crohn’s disease	(18)
Spontaneous	TNF^ΔARE^	Ileitis develops spontaneously due to increased stability and production of TNF due to the deletion of the *TNF* AU-rich elements.	Develops patchy inflammation in the ileum and proximal colon – similar to Crohn’s disease, Neutralization of TNF alleviates inflammation, 100% penetrance of disease in this strain of mice	(19, 20)
Infectious	*Citrobacter rodentium*	*C. rodentium* attaches to the intestinal epithelial cells driving the cytokine production and activation of both innate and adaptive immune responses	Models human infection with enteropathogenic *Escherichia coli*, Allows the researcher to understand host-pathogen interactions in the context of intestinal inflammation	(21)
Infectious	*Helicobacter hepaticus*	*H*. *hepaticus* colonizes the colon of mice. In the absence of IL-10 signaling, colitis is characterized by the increased expression of IFNγ and IL-17A	A model for studying the role of IL-10, IL-23, IL-17A, and the innate immune system in the development of intestinal inflammation	(22, 23)

Multimodal single-cell technologies, such as single cell RNA-sequencing (scRNA-seq), have allowed for in-depth analysis and a deeper understanding of tissue architecture and function at individual-cell high-resolution (24). ScRNA-seq has been instrumental to our understanding of immunology and autoimmune diseases, where complex cellular heterogeneity and interactions between immune cells, stromal cells, and epithelial cells are paramount for disease progression. These technologies have enabled an improved understanding of human IBD by identifying novel or rare cell types that were previously undiscovered and how their abundance or function changes during intestinal inflammation may be a driver of disease (25). While scRNA-seq technology has been applied to numerous UC and CD cohorts to better understand the complex biology between immune cells and non-hematopoietic cells, this technology has only begun to be applied to pre-clinical models of IBD for comparative studies with the human condition.

While studies have enriched specific cell populations from pre-clinical models of IBD and characterized these cells by scRNA-seq (26–28), there are gaps in the knowledge of the entire makeup of the inflamed tissue. In this review, we have summarized the key findings from a longitudinal scRNA-seq study that analyzed immune cells, stromal cells, and epithelial cells. Furthermore, we highlight the similarities and differences between scRNA-seq in murine models compared to human IBD. Finally, we emphasize the need for utilizing scRNA-seq technology to find suitable translational models based on the therapeutic target.

## A longitudinal scRNA-seq atlas in experimental colitis

While there are other studies utilizing scRNA-seq in enriched cell populations from pre-clinical models, highlighted below is one study that has applied scRNA-seq technology to comprehensively describe the landscape of the epithelial cell, stromal cell, and immune cell populations during the onset and resolution of intestinal inflammation (29). Ho et al. subjected wild-type mice to 1.5% dextran sodium sulfate (DSS) drinking water for 6 days, then placed mice on regular drinking water for the remainder of the study. Colon tissue was collected from mice on days 0, 3, 6, 9, 12, and 15 in biological triplicates. ScRNA-seq was performed on viable cells and 14,624 cells were profiled. ScRNA-seq analysis identified 15 clusters including: mononuclear phagocytes (*Il1b*, *C1qa*, and *C1qb*), epithelial: enteroendocrine cells (*Scgn* and *Pcsk1n*), epithelial: absorptive and secretory cells (*Muc2*, *Spink4*, *Lypd8*, and *Elf3*), endothelial cells (*Pecam1* and *Flt1*), lymphatic cells (*Lyve1*), stromal cells (*Col1a1*, *Pdgfra*, and *Spon2*), two myofibroblast clusters (*Acta2* and *Myh11*), interstitial cells of Cajal (*Ano1* and *Kit*), enteric glial cells (*S100b*), T cells (*Cd3d* and *Cd3g*), plasma cells (*Igha* and *Mzb1*), plasmacytoid dendritic cells (*Siglech* and *Ccr9*), B cells (*Cd19* and *Cd22*), and granulocytes (*Cd14*, *S100a8*, and *S100a9*) (29).

To assess changes in the cellular composition, the numbers of cells in every cluster were counted at each sampling time point. Epithelial cells, stromal cells, myofibroblasts, interstitial cells of Cajal, and enteric glial cells were reduced in number with inflammation and did not repopulate during the resolution phase. In contrast, the numbers of granulocytes and B cells increased with inflammation and did not return to homeostatic numbers measured at steady state. Mononuclear phagocytes peaked in number 6 days after DSS administration and returned to baseline at the end of the study after the removal of DSS drinking water. Lastly, plasma cells decreased at days 6 and 9, but recovered in total numbers by the end of the resolution phase on drinking water at day 12. Furthermore, differentially expressed gene (DEG) analysis demonstrated phenotypic changes in mononuclear phagocytes and stromal cell clusters across all time points. The authors selected 96 “IBD risk genes” to assess how the DEGs identified in their longitudinal scRNA-seq study correlated with human IBD, 79 of the selected 96 human IBD risk genes were expressed at one or more time points and in one or more of the dysregulated cell clusters, suggesting that a dynamic expression pattern is under regulation during intestinal inflammation (29). One example is *Ifng*, which was found to be expressed during the time points associated with inflammation within the T cell cluster (29).

While scRNA-seq has been seldom applied to pre-clinical models of IBD, scRNA-seq has been more readily applied to multiple CD and UC patient cohorts to identify novel cell types, cell-cell interactions, and to generate new hypotheses of IBD pathogenesis. We will discuss in the next section the differences that have been found in human IBD using scRNA-seq in the epithelial cell, stromal cell and immune cell compartments compared to the limited murine studies including but not limited to the study described above.

## scRNA-seq in human IBD by cell type and differences observed in murine models

### Intestinal epithelial cells

The lining of the gastrointestinal tract (GI) is composed of diverse populations of intestinal epithelial cell lineages with specialized functions. These epithelial cells are derived from a common stem-cell precursor, which expresses *LGR5* (30). Intestinal epithelial cells contribute to intestinal homeostasis by maintaining barrier function and secreting mediators in response to the microbiota and underlying immune cells in the lamina propria.

The first scRNA-seq study analyzing human colonic epithelial cells from healthy volunteers or patients with UC, sampled from clinically inflamed and noninflamed mucosa, was published by Parikh et al. in 2019 (31). Visualization of scRNA-seq data identified 10 clusters of intestinal epithelial cells including undifferentiated cells, absorptive colonocytes, goblet cells, and unique enteroendocrine cell populations including L-cells, enterochromaffin cells, and precursor-like cells, and 5 clusters of stem cells. In UC, two additional clusters were identified representing inflammation-associated goblet cells and intraepithelial immune cells. Not only did this analysis provide a comprehensive landscape of the epithelial cells that are present in the healthy colon, but also identified their dysregulation in inflamed UC. One of these subsets were “BEST4/OTOP2” colonocytes. *BEST4* encodes a calcium-sensitive chloride channel, while *OTOP2* encodes a proton-conducting ion channel. These findings highlighted the power of scRNA-seq technology in identifying novel cell populations which could contribute to intestinal homeostasis and/or inflammation. Another prominent cell type linked to disease pathogenesis and barrier function are goblet cells. Goblet cells are mucus-secreting epithelial cells that are critical for the maintenance of barrier function and prevention of bacterial translocation. In IBD both their function and numbers become dysregulated (32). ScRNA-seq analysis of healthy human and UC colonic tissue revealed a distinct cluster of inflammation-associated goblet cells that had decreased expression of *WFDC2* (31), a gene encoding an anti-protease enzyme (33). To explore the significance of the loss of *WFDC2* expression in goblet cells in intestinal homeostasis, *Wfdc2* ^+/-^ mice were generated. These mice had abnormalities in colonic epithelial intercellular tight junction proteins, mild-to-modest epithelial cell hyperplasia, immune cell infiltration into the tissue, and proximity of gram-negative and gram-positive bacteria to the colonic epithelium compared to littermate controls (31). Although these mice were not subjected to an induced model of colitis, this study demonstrates how findings from human scRNA-seq samples can be translated in an *in vivo* setting of gastrointestinal tract dysregulation.

These novel populations become critically important in IBD as breakdown in intestinal barrier function is a hallmark phenotype. There has been great interest in underpinning the mechanisms and contributions of epithelial subsets in this loss of barrier function and to target barrier regeneration with novel therapeutics. In other studies, the authors have performed scRNA-seq analysis on healthy and CD or UC cohorts to assess the transcriptomic changes in epithelial cell subsets. One common finding amongst these scRNA-seq analyses is a depletion of BEST4^+^/OTOP2^+^ colonocytes in UC and CD compared to healthy controls (31, 34, 35). While BEST4-expressing colonocytes are present in the human GI tract, this cell population is absent in the murine gut (36).

The primary function of microfold (M) cells is the transport of antigens from the lumen of the intestine to gut-associated lymphoid tissues such as Peyer’s patches (37). ScRNA-seq uncovered an expansion of M cells in UC patients compared to healthy controls (34). Furthermore, these cells expressed elevated levels of *CCL20* and *CCL23*, important chemokines driving immune cell recruitment into the inflamed tissue. The expansion of M cells, detected by immunohistochemistry, held true in a DSS colitis model and treatment with an anti-TNF antibody abrogated the expansion of M cells in the inflamed tissue in mice, suggesting the pro-inflammatory milieu in IBD can impact the differentiation of intestinal epithelial cells during disease (38).

In addition to aiding in our understanding of cell populations in barrier breakdown at the single cell level, scRNA-seq has contributed to identifying novel biological pathways in epithelial cells. Gasdermin-D (GSDM) plays a pivotal role in driving cell death through the pore-forming activity of the protein’s N-terminus and has recently been discovered to be required for IL-1β release (39, 40). ScRNA-seq of colonic tissue between healthy and UC patients revealed that secretory and absorptive progenitors, as well as colonocytes, from UC patients express elevated levels of *GSDM* (31, 41). This finding highlights a new mechanism by which GSDM-mediated IL-1β release by intestinal epithelial cells could sustain intestinal inflammation. In yet another example of how scRNA-seq translated into an *in vivo* model, *Gsdmd^+/-^* mice are protected from DSS-induced colitis as indicated by a decreased infiltration of neutrophils, macrophages, and T cells, and decreased expression of inflammatory cytokines and chemokines (41). This study reiterates the power of scRNA-seq in identifying pathways in previously underappreciated cell populations that are critical for inflammation.

In the longitudinal DSS study described above by Ho et al. (29), upon administration of DSS, enteroendocrine and both absorptive and secretory epithelia cell clusters were reduced. Thus, applying scRNA-seq to additional pre-clinical IBD models would be advantageous to find a suitable model to translate scRNA-seq findings in epithelial cells in human IBD.

### Stromal cells

Stromal cells are part of the mesenchymal compartment of the intestine, and represent a distinctive heterogeneous population comprised of non-hematopoietic, non-epithelial cell types (42). Mesenchymal cells regulate homeostasis by promoting extracellular matrix (ECM) turnover through the secretion of ECM factors, such as collagen and glycoproteins, self-renewal of the intestinal epithelial barrier through WNT/β-catenin signaling and colon regeneration through production of prostaglandins (43). However, in the context of inflammation these pathways are subverted into inflammatory processes. During intestinal inflammation, dysregulation of Wnt signaling leads to impaired wound healing characterized by the activation of fibroblasts and myofibroblasts, the accumulation of collagen-rich ECM, culminating in fibrosis. Additionally, TGF-β1 signaling in fibroblasts leads to their transition to an activated phenotype, marked by an increase in ECM production, leading to fibrosis. ScRNA-seq has played a significant role in elucidating the heterogeneous function of stromal cells and highlighted novel ‘cross-talks’ with other cells in the intestinal microenvironment. In the first report using unbiased single-cell profiling of over 16,500 human colonic mesenchymal cells from healthy and treatment-naïve UC patients, Kinchen and colleagues have shown how the colonic mesenchyme compartment remodels in the context of IBD (44). Specifically, the authors identified key stromal cell populations involved in immune homeostasis in intestinal inflammation. They identified a distinct cluster found near the epithelial crypts marked by the expression of *SOX6*, *F3* (CD142) and *WTN* genes, which was important in mediating epithelial cell self-renewal in physiological conditions. Interestingly, scRNA-seq of stromal cells from the DSS colitis model revealed similarities between human and mouse stromal cell clusters, with *SOX6* being highly conserved between the two (44).

ScRNA-seq of UC colonic mesenchyme revealed changes in gene expression and cell composition within the stromal compartment (44). Single-cell analysis identified 12 distinct clusters, with a significant expansion of a cluster marked by genes involved in response to TNF and leukocyte migration. Highly ranked markers included lymphocyte trafficking chemokines, such as *CCL19* and *CCL21*; T cell co-stimulatory TNF-superfamily ligand (*TNFSF14*/*LIGHT*) and MHC II invariant chain (CD74); CD24; IL-33 and Lysyl oxidases. In UC, the enrichment of this cluster was associated with a decrease in the *SOX6* expressing cluster in inflamed tissues, further highlighting the heterogeneous nature of the stromal-driven responses. Thus, this study revealed a divergent response in the context of disease, marked by an increase in pro-inflammatory fibroblasts and a reduction in the stromal subsets associated with epithelial self-renewal and resolution of disease. The role of stromal cells in IBD was further validated in the DSS model of colitis (44). ScRNA-seq analysis revealed high expression of *Lox* and *LoxI1* in the mesenchymal populations. Lastly, the authors investigated whether blockade of this pathway could ameliorate DSS. The Lox family of lysyl oxidases generate oxidative stress responses, disturbing the redox balance within the tissues, which in turn mediate recruitment of inflammatory mediators, thus sustaining IBD pathogenicity. Indeed, administration of Lox/LoxI1 inhibitor, β-aminopropionitrile, improved the clinical score of mice receiving DSS drinking water (44).

Kong et al. profiled 720,633 cells from the terminal ileum and colon of CD and non-IBD donors by scRNA-seq (45). The authors identified *CHMP1A, TBX3, and RNF168* as disease-associated genes which are affiliated with myofibroblast activation (45). To validate the roles of these three genes in fibrosis development, the authors generated and pooled siRNA oligos to knockdown *CHMP1A, TBX3, and RNF168* in normal human intestinal fibroblasts. Knockdown of *CHMP1A, TBX3, and RNF168* significantly impaired TGF-β-driven collagen gene expression (45).

To gain a better understanding of the complexity of the fibroblast cellular networks and how they underly stricture formation in CD, Mukherjee and colleagues have generated the first scRNA-seq atlas of strictured bowel from CD patients (46). Specifically, scRNA-seq was performed on CD resections containing non-involved, inflamed non-strictured and strictured segments, as well as non-IBD bowel segments. ScRNA-seq analysis revealed significant differences in the cellular composition between non-inflamed, inflamed and strictured segments. Comparison between non-strictured and strictured segments revealed significant changes in lymphocytes, smooth muscle cells, endothelial cells, fibroblasts and myeloid cells within the epithelial and lamina propria fractions. Additionally, the strictures were highly heterogenous with regards to cell composition, with 19 distinct cell types being identified, further clustered into fibroblasts, smooth muscle and endothelial populations (46).

Further analysis of the fibroblast compartment revealed six fibroblast populations enriched in CD strictures as opposed to non-strictured segments (46). These fibroblasts were characterized by an inflammatory phenotype, marked by the expression of pro-fibrotic markers involved in TGF-β signaling, macrophage differentiation and ECM production. However, the predominant cluster in these strictures were the MMP^+^/WNT5A^+^ fibroblasts, which were previously found to be enriched in the mucosa of patients with UC resistant to anti-TNF therapy (46). Among the populations within the stromal niche, CXCL14^+^ fibroblasts were the main contributors to transcriptional changes in strictures. These fibroblasts were associated with increased ECM production and CXCL14 displayed pro-fibrotic functions (46).

More importantly, the authors identified Cadherin11 (*CDH11*) as the only cell surface receptor co-expressed in both MMP^+^/WNT5A^+^ and CXCL14^+^ fibroblasts. Interestingly, *CDH11* expression was shown to increase in CD strictures. The therapeutic potential of targeting CDH11 was shown in a DSS-induced fibrosis model of colitis in *Cdh11* knockout mice (46). In this model, mice are subjected to two cycles of DSS drinking water followed by a recovery period with normal drinking water. Despite no differences in inflammation being observed in *Cdh11* knockout mice compared to wild-type controls, *Cdh11*-knockout mice were protected from fibrosis development. Similarly, administration of an anti-Cdh11 mAb ameliorated DSS-driven fibrosis. In a longitudinal scRNA-seq analysis in the DSS model, Ho et al. observed that stromal cell clusters had strong influences on other clusters, highlighting their potential role in intestinal inflammation (29). Looking closely at these interactions, the authors identified relevant genes during the recovery phase, with the highest expression observed in *Serpina3n*, a serine protease inhibitor. To validate these observations, the authors examined the progression of DSS colitis in a *Serpina3n* knockout mouse. Although the disease progression was similar to wild-type mice, the knockout mice recovered much faster. Furthermore, the authors examined the mechanisms by which Serpina3n ameliorated DSS-induced intestinal inflammation. Using scRNA-seq on colon treated with Serpina3n protein, they observed that genes associated with ECM organization and cytokine mediated signaling pathways were enriched in stromal cells. Additionally, there was a decrease in inflammatory interactions and in the number of neutrophils, warranting the therapeutic potential of Serpina3n to accelerate recovery from intestinal inflammation. Overall, scRNA-seq has been crucial in characterizing the heterogeneity of the stromal population, as well as their interactions with the immune compartment in IBD. Alternatively, applying this technology to preclinical models of IBD could help uncover important mechanisms underlying resistance to current therapies. To address this, West and colleagues used transcriptomic approaches to uncover relevant genes associated with anti-TNF resistance. Oncostatin M (OSM) is expressed by stromal cells and was identified as a potential biomarker associated with anti-TNF failure (47). This observation was validated in a mouse model of IBD driven by oral *Helicobacter hepaticus* infection and anti-IL-10R blocking antibody, which mirrors the T cell-dependent pathology driving resistance to anti-TNF therapy. Using this model, treatment with an oncostatin receptor-Fc significantly attenuated colitis (47). In a more recent study, bulk and single-cell transcriptomics have been applied to inflamed tissue from three IBD patients’ cohorts undergoing surgical resection (48). The authors observed similar gene signatures from therapy non-responders, marked by genes indicating high neutrophil infiltration and activation of fibroblasts at sites of deep ulceration. Further analysis revealed important neutrophil-attractant signatures in fibroblasts, marked by an increase in IL-1R signaling, suggesting that this pathway is important in the inflammatory fibroblast/neutrophil recruitment in patients that are nonresponsive to current therapy (48).

### Immune cells

#### Monocytes/macrophages and dendritic cells

In active IBD, macrophages secrete pro-inflammatory cytokines and react to commensal bacteria, with CD exhibiting aggregates of macrophages and other inflammatory cells (granulomas) associated with higher resection risk (49). IBD susceptibility involves various genetic loci linked to monocyte and macrophage function, including *NOD2*, *ATG16L1*, and *IRGM* (50–52). In 2019, two scRNA-seq studies explored intestinal macrophages in CD and UC. One scRNA-seq analysis of 11 ileal CD patients revealed resident and inflammatory macrophage subtypes. The resident subtype expressed *C1Qs*, *CSF1R*, *MAFB*, and *MRC1*, while inflammatory macrophages showed elevated levels of chemokines (*CXCL2*, *CXCL3*, *CXCL8*) and cytokines (*IL23*, *IL6*, *IL1B*, *IL1A*, *TNF*, *OSM*) Notably, pro-inflammatory interactions between non-epithelial stromal cells and macrophage-derived oncostatin-M (OSM) were found within the inflamed intestine, linked to anti-TNF-resistant CD (53). The second scRNA-seq study conducted on 18 UC patients revealed the presence of an inflammatory monocyte cluster with prominent levels of *OSM* expression that were associated with disease progression and resistance to anti-TNF treatment. A macrophage cluster was also identified that closely interacts with B cells and T cells. This macrophage cluster exhibited an enrichment of genes linked to IBD susceptibility, such as *GPR65*, *ADCY7*, *PTGER4*, *PTPRC*, and *SH2B3* (34). A very recent scRNA-seq study provided a therapeutic map for CD and UC after anti-TNF therapy in a longitudinal manner (54). Importantly, the presence of a granuloma signature, characterized by C1Q^high^ IL1B^low^ resident macrophage cells, was specifically associated with non-responders in CD. Moreover, genes associated with an inflammatory monocyte profile (*S100A9, S100A12, FTH1, IL1RN*) showed higher levels in non-responders but were reduced in responders. The latest study utilizing scRNA-seq and spatial analysis (CosMx Spatial Molecular Imaging) found that resident macrophages were present in both healthy and active IBD patients (55). Notably, inflammatory macrophages displayed patient-specific adaptation, characterized by an alternative activation pattern featuring the expression of EGFR ligands, *NRG1*, *HBEGF*, *CLEC10A*, and *ASGR1*, differing from the conventional M1 signature. The study also suggested a potential role of interactions between M2 or inflammation-dependent associated macrophages (representing the predominant inflammation-dependent macrophage state) and inflammatory fibroblasts in contributing to disease pathophysiology.

Through scRNA-seq studies in mice (56–61), we have gained insights into the diverse inflammatory roles of gut macrophages. In the DSS-induced colitis study, the abundance of *Il1b^+^ C1qa^+^ C1qb^+^* macrophages increased in diseased animals, peaking post DSS administration, then returning to baseline during water administration (29). The mouse single-cell findings align with how intestinal cues influence transcription factors shaping macrophage subsets. The recent single-cell investigation using the *Helicobacter hepaticus*-induced colitis model, highlights interferon regulatory factor 5 (IRF5)’s role in mucosal macrophage differentiation. *Irf5* knockout hindered CD11c^+^ mucosal macrophage formation, unveiling IRF5’s contribution to pro-inflammatory CD11c^+^F4/80^+^ macrophages during inflammation, driving colitis development (60). IRF5’s link to inflammatory macrophages and genetic risk factors for UC and CD underlines these mouse findings’ translational relevance to human IBD (62).

Dendritic cells (DCs) are antigen presenting cells that bridge both innate and adaptive immune responses. In the intestine, DCs continuously sample antigens by extending processes through the epithelial cells and glycocalyx layers into the lumen, or by remaining in the lamina propria or Peyer’s patches to process antigens that may have translocated (63). While multiple scRNA-seq studies in humans have begun to showcase the importance of different DC subsets in driving IBD pathogenesis, scRNA-seq studies in mouse models of IBD lag, specifically in the inclusion of diverse DC subsets, such as plasmacytoid DCs (pDCs), conventional DCs (cDCs), and monocyte-derived DCs (moDCs) (34, 45, 53, 54, 64). Corbin et al. used *Helicobacter hepaticus*-induced colitis coupled with an anti-IL-10R mAb in *Irf5^-/-^* mice and found the frequencies of DC populations during ongoing inflammation remained unchanged compared to steady-state (60). ScRNA-seq analysis on mononuclear phagocytes taken from the inflamed colonic lamina propria of CX3CR1^+^ wild-type and *Irf5^-/-^* mice. Within the four clusters of DCs, all lacked *Cd64* expression, but included *Flt3, Cd11c*, and the DC-specific MHCII genes *H2-DMb2* and *H2-Oa.* DC2-like cells were identified by *Sirpa, Kmo, Cd209a*, and *Cd7* expression. The strong expression profile of *Pu.1* but low *Flt3* expression within these cells from these clusters suggested some may be moDCs. The other two clusters were likely representative of cDC1s (termed “*Xcr1* DC” and expressing *Xcr1* ^high^ *Irf8* ^high^ *Sirpa* ^low^) and a smaller group of *Ccr7^+^* migratory DCs. Ho et al. also identified by scRNA-seq that pDCs decrease in number shortly after DSS exposure, however, the numbers stabilize over the resolution phase (29). Overall, this experiment showed that the heterogeneity of DCs could be identified and classified in the inflamed colonic tissue from these mice using scRNA-seq. The scarcity of murine single-cell studies necessitates further scRNA-seq in IBD models for a better understanding of these cells’ pivotal roles.

#### CD4^+^ T cells

CD4^+^ T cell function in the intestine can be broadly categorized as effector (pro-inflammatory) or regulatory (anti-inflammatory). Regulatory T cells (Tregs) are CD4^+^ T cells that are critical for tolerance and dampening excessive immune responses against dietary and microbial antigens, an integral mechanism for maintaining intestinal homeostasis (65). In IBD, this balance of anti-inflammatory Treg function is lost and exuberant pro-inflammatory T cell responses against the commensal microbes drives and sustains chronic inflammation. In the paragraphs below, we will mainly highlight Treg and TH17 biology (63).

## Regulatory T cells

ScRNA-seq studies have been instrumental in advancing our understanding of the complex cellular heterogeneity and gene expression patterns of intestinal Treg cells in UC and CD. ScRNA-seq uncovered previously unidentified FOXP3^+^ cell subtypes involved in IBD. In a related study, Corridoni et al. (66) utilized multi-modal scRNA-seq to examine colonic CD8^+^ T cells in healthy individuals and those with UC. Interestingly, they noted an elevated presence of double-positive CD4^+^ CD8^+^ FOXP3^+^ T cells in UC patients. Smillie et al. (34) noted heightened levels of CD8^+^IL-17^+^ T cells and Tregs in inflamed UC tissues. They observed a TNF expression shift towards Tregs during inflammation. The study proposed that TNF^+^ Treg cells could significantly influence IBD progression, potentially causing resistance to TNF antibodies and playing a crucial role in CD8^+^ T cell adaptability. In healthy tissue, Tregs represented 1% of TNF expression, but this increased to over 14% during inflammation. Interestingly, in another study by Devlin et al. 67) found increased FOXP3^+^/BATF^+^ Treg cells in inflamed UC patients. They used scRNA-seq on CD45^+^ hematopoietic cells from UC patients with and without ileal pouch-anal anastomosis. These Tregs were enriched in inflamed UC and pouchitis samples, suggesting a role for BATF in their recruitment during inflammation. ScRNA-seq conducted by Boland et al. (68) revealed transcriptionally distinct Tregs expressing *ZEB2* in UC. Among Treg gene profiles, 288 genes differed between healthy individuals and UC patients, including *SATB1*, *KLF2*, *MYC*, and *ITGB1*, associated with Treg function. Notably, *ZEB2*, linked to CD8^+^ T cell function, exhibited high expression in UC-related Treg cells. The reduction of ZEB2 enhanced suppressive function in murine Treg cells.

Huang et al. (69) analyzed initial diagnosis colon biopsies from healthy individuals and CD/UC patients, finding enriched Treg cells (slightly more in CD) and CD8^+^GZMK^+^ effector memory T cells in both conditions. Additionally, Jaeger et al. (70) used scRNA-seq and multi-parameter flow cytometry or mass cytometry to examine CD patients’ inflamed terminal ileum tissue. Inflammatory tissue IEL showed reduced Tregs, CD8^+^T, γδT and TFH but elevated TH17 cells. Lamina propria (LP) analysis revealed decreased Tregs and TFH cells, while CD8^+^T cells and TH17 cells increased. Tregs in LP were enriched in *GPX1* and *GLRX*, associated with oxidative stress protection via FOXP3. *GPX1* expression in LP Tregs hinted at activated anti-oxidative pathways, pertinent to CD due to *GPX1* gene’s CD risk connections. Martin et al. (53) examined ileal samples from 13 CD patients at the single-cell level. In their pursuit of unraveling the cellular profile linked to resistance against anti-TNF drug therapy, they observed an abundance of Tregs with elevated IL-10 levels, along with low IL-17A expression within inflamed ileal lesions. Additionally, Treg populations have been detected in CD and UC patients by Mitsialis et al. (71). They observed pro-inflammatory memory-like IL17A^+^ Treg subsets expanded uniquely in active UC. Among IBD-enriched memory Tregs, one group was FOXP3^+^, while another was FOXP3^low^ with strong pro-inflammatory cytokine expression (IFNG^+^TNF^+^IL17A^+/–^), indicating altered Treg function in IBD. Additionally, they observed an unusual expansion of novel *IL1B^+^* Tregs in peripheral active CD compared to inactive CD and active UC. Furthermore, in a recent development by Nie et al., a computational tool called “scIBD” provided an integrated assessment of scRNA-seq data associated with IBD. This platform enabled the recognition of different cell types and IBD-associated genes. The meta-analysis corroborated the greater prevalence of Tregs in both UC and CD compared to healthy tissues, consistent with prior findings (72).

The study by Miragaia et al. (73) conducted single-cell analysis to comparatively investigate human and murine colon Treg populations. In mice, three distinct Treg subpopulations were identified: non-lymphoid tissue (NLT), suppressive, and lymphoid tissue-like (LT). NLT Tregs displayed a GATA3^+^-subpopulation phenotype, expressing *Gata3*, *Nrp1*, *Areg*, *Il1rl1*, and *Ikzf2*, while suppressive Tregs resembled the peripherally derived RORγt^+^-subpopulation. LT-like Tregs exhibited LT-associated gene expression (*Sell*, *Ccr7*, *Tcf7*, *Bcl2*) and fewer NLT-associated genes (*Klrg1*, *Cd44*, *Icos*, *Rora*, *Tnfrsf9*, *Itgae*), signifying functional diversity. Notably, 17 human-mouse colon Treg markers overlapped, including *Tnfrsf4*, *Lgals1*, *Srgn*, *Cxcr6*, *Maf*, and *Ikzf3*, indicating conserved roles. Paralogous genes demonstrated inter-organism expression pattern substitutions (e.g., *Pim1*-*Pim2*), hinting at the evolution of cellular communication pathways. Despite interspecies disparities, the study underscores the conservation of the TNFRSF-NF-κB-pathway between mice and humans (73). Surprisingly, the scRNA-seq study mentioned earlier, which focused on the murine DSS model (29), did not identify Tregs in any of the collected time course samples. However, a very recent preprint (74) took a different approach. Using MERFISH, a method for spatially resolved single-cell transcriptome profiling, they studied spatial and cellular changes during gut inflammation initiation and recovery in a DSS model. In day 9 samples, corresponding to peak inflammation after DSS administration, mucosal remodeling was observed, along with the appearance of diverse cell populations, including Tregs. Thus, the limited investigation of murine Tregs at the single-cell level underscores the need for further scRNA-seq studies on murine IBD models to gain a deeper understanding of the roles played by this essential cell population.

## TH17 cells

Naïve CD4^+^ T cell polarization towards an effector phenotype requires signals from antigen-presentation, co-stimulation, and cytokines in the local tissue environment. *In vitro* polarization of human CD4^+^ T cells towards a TH17 phenotype requires TGF-β, IL-1β, IL-6, IL-23 and the expression of RAR-related orphan receptor g (RORγT) (75). Polarized TH17 cells secrete IL-17A, IL-17F, and IL-22 – cytokines which are associated with the development of inflammation. While not required for TH17 differentiation, IL-23 signaling, through the IL-23 receptor complex, promotes RORγT stability and the acquisition of a pathogenic phenotype in these cells. IL-23 is upregulated in numerous autoimmune diseases, including IBD (76). Furthermore, genome-wide association studies have identified *IL23R* as a genetic susceptibility locus for IBD. A key role for IL-23 in IBD pathogenesis has been highlighted by the clinical efficacy of anti-IL-23 biologics (77–79).

Jaeger et al. used scRNA-seq analysis to compare T cells from intra epithelial lymphocytes (IELs) and the lamina propria (LP) found in the terminal ileum of adults with severe CD to measure heterogeneity in T cell lineages and subsets (70). There was a marked increase in pro-inflammatory, activated CD39^+^CCR6^+^ and CD39^+^CD4^+^ TH17 cells but decreased CD8^+^T, γδT, TFH and Treg cells in the IEL compartment of CD patients, demonstrating that loss of balance between TH17 and Treg cells plays an important role in disease progression. Within IELs, they identified unique T cell clusters, including one with NKp30^+^γδT cells expressing RORγt and producing IL-26 upon NKp30 engagement. Some of these T cell subsets expressed TH17 markers including *RORC, IL23R, IL22*, *CXCR4*, and *IL26*. While these RORγt^+^ γδ T cells have been shown and described in mouse models (80, 81), Jaeger et al. identified this subset of γδ T cells in the IEL of humans. γδ T cells can constitute as much as 40% of the IELS in the intestine. These cells can have cytotoxic, immunoregulatory, and tissue repair functions within the intestinal mucosa (82). However, the role of human γδ T cells in CD remains to be fully elucidated, with multiple studies showing either a decrease or increase in number in the blood or inflamed tissue (82). Similar analysis in the LP found a biased TH17/Treg ratio in CD. The CD39^+^ TH17 cells that were found within the IEL may prove to be a double-edged sword, as their expression of *GZMB* and *CCL4* could make them pathogenic and able to recruit other inflammatory cell types to increase epithelial damage (70). Conversely, their production of IL-17 and IL-26 may enhance epithelial barrier integrity and protection (70). Overall, this study showed an altered spatial distribution of T cell subsets between the two cellular compartments, with TH17s in the LP bearing more markers of quiescence (i.e., CXCR4 and CD39) than in the IEL compartment that may correlate with transmural inflammation during IBD pathogenesis (70).

An atlas of over 350,000 cells from the colonic mucosa of patients with and without UC was used to reveal 51 cell subsets, including epithelial, stromal, and immune cells (34). When reviewing T cells, several CD4^+^ T cell subsets showed increased expression of *IL17A*, showcasing an expansion of TH17 cells with pro-inflammatory phenotypes. However, the CD8^+^ T cell subsets showed the largest induction on *IL17A*. Using an *in-situ* analysis, the authors found CD8 co-expressed on both CD4^-^ and CD4^+^ cells. These CD4^+^CD8^+^IL17^+^ T cells have been largely uncharacterized, however, these cells correlated with activated cytotoxic programs and genetic signatures conducive to TH17 pathogenicity and tissue damage (e.g., *RBPJ* and *IL23R*) that have been reported in mice (83). Kong et al. also showed *IL23R* to be an IBD risk gene in cells taken from the colon and terminal ileum of patients with CD, but they showed the greatest mean expression to be in innate lymphoid cells (ILCs) in both tissue types (45).

A recent study in mice showed that colonic immunopathology driven by Red 40, and IL-23 may not actually depend on these classical TH17 responses; as IL-17A and IL-17F blockade did not prevent colitis development in mice that conditionally expressed *IL23* in CX3CR1-positive myeloid cells (84). Additionally, they sought to define this mechanism further. They used the T cell transfer colitis model, an invaluable model to dissect pathogenic and protective mechanisms of CD4^+^ T cells (11) and scRNA-seq to identify a new population of IFNγ-secreting CD4^+^ cytotoxic T lymphocytes that were induced by IL-23 and the food dye Red 40 to promote colitis pathogenesis (27). Without IFNγ, IL-23 and Red 40 did not induce progressive colitis development and prevented colonic epithelial cell death, showcasing perhaps a specialized relationship between IL-23, IL-17, IFNγ, and overall TH17 signaling in the development of IBD in mice (27).

In a longitudinal DSS mouse model, Ho et al. looked at how cell phenotypes changed during inflammation (29). Using a pseudotime analysis, significant changes were observed in T cell subsets (which expressed *Cd3d* and *Cd3g*). The IBD risk gene *Ifng* was expressed over the entire DSS-induced disease time course in the T cell cluster but only at day 6 in stromal cells, indicating subtle dynamic phenotypic changes in different cell types. Capturing these subtle changes over time in a human model is difficult, exemplifying how useful mouse models of IBD can be, especially when paired with scRNA-seq analysis.

### CD8^+^ T cells

CD8^+^ T cells are a cytotoxic T cell subset that drive cell-death through numerous pathways including perforin and granzyme release and by expressing FAS ligand (85). CD8^+^ T cells are typically in lower abundance in the intestine at steady state compared to CD4^+^ T cells (86). Despite numerous evidences that IBD pathology is associated with exuberant CD4^+^ T cell responses, recent scRNA-seq data revealed that CD8^+^ T cells also play a role in IBD pathogenesis (25, 66, 68).

T cells sorted from the IELs of healthy donors and CD patients revealed different CD8^+^ clusters (70). One of these clusters expressed genes associated with an effector phenotype (*KLRG1*, *GZMB*, *GZMK*, and *IFNG*). Whereas, the other CD8^+^ cluster highly expressed the tissue residency marker *ITGAE*. When these cells were analyzed further using a new clustering strategy, this highlighted the expression of *IL7R*, *IL2* and *TCF7*, genes associated with a memory phenotype. The authors hypothesized that the resident memory CD8^+^ T cell cluster might gain cytotoxic and antimicrobial functions via S100A family members during inflammation. In the same study, scRNA-seq of CD8^+^ T cells in the LP identified three clusters: effector CD8^+^ T cells (*KLRG1*, *EOMES*, *GZMB*), a second group expressing CD160 and *ITGA1*, and a third group expressing NK cell receptors such as *KLRC1*, *KLRD1*, and *ENTPD1* (70). In summary, similar and unique CD8^+^ T cell clusters were identified in the IEL and LP compartments of CD patients.

A second scRNA-seq analysis of colonic T cells revealed that CD8^+^ and γδ T cells both expressed *ENTPD1* (64). This gene codes an ectonucleotidase that converts ATP and ADP to AMP. In colonic CD8^+^ T cells from pediatric cases of colitis, decreased *ENTPD1* expression was observed in association with a defective cyclic AMP (cAMP) pathway. Mechanistically, the reduction of cAMP is believed to result in platelet aggregation and hyperinflammation. The authors then used DSS-induced acute colitis mouse model to test the effect of dipyrimodole, a phosphodiesterase (PDE) inhibitor. PDE is an enzyme the degrades and inactivates cAMP and cGMP, therefore, inhibiting PDE will increase cAMP levels *in vivo*. Administering mice dipyrimodole increased the colon length, prevented body weight loss, improved barrier function, and crypt structure in the DSS treated mice. As a result, they demonstrated increased expression of *Entpd1* and elevated cAMP levels also influenced platelet aggregation and TNFα production, linking the findings observed in a pre-clinical model of colitis back to human IBD (64).

In yet another study, scRNA-seq of CD8^+^ T cells from dissociated colonic tissues of UC patients, identified tissue resident (*ITGAE* and *DUSP4*), naïve (*SELL*, *CCR7*, and *TCF7*) double positive CD8^+^CD4^+^ (*GZMA*, *RORA*, and *CCR6*) and increased numbers of *IL26* expressing T cells (66). Although *IL26* is not expressed in mice, human IL-26 can signal through the IL-10RB and IL-20RA heterodimeric receptor in mice (87). Deeper analysis of the *IL26*-expressing CD8^+^ T cells displayed both an ILC3 signature (*RORC*, *CKIT and AHR*), a TH17 signature, and several exhaustion markers (*PDCD1, HAVCR2, and CTLA4*). Interestingly, these *IL26* expressing CD8^+^ T cells co-expressed *IL23R* and *IL17A*, which has also been shown in a recent longitudinal human single cell therapeutic atlas of IBD (54). To further assess the role of *IL26* in UC, the authors subjected humanized *IL26* transgenic mice to DSS-induced colitis and tested the efficacy of an anti-IL-26 monoclonal antibody. Using these tools, the authors demonstrated that IL-26 has a protective role in the acute model of colitis in mice; however, whether IL-26 has the same protective effect under chronic inflammation conditions remains to be elucidated (66).

While typically associated with protecting the host from infection, a pathogenic role for resident memory CD8^+^ T cells (Trm) in autoimmune diseases have become appreciated (53, 68, 88). Boland and colleagues identified four CD8^+^ Trm clusters, expressing high levels of *CD69, ITGAE, CD101, CCR6, ITGA1* and lower levels of *KLF2* and *S1PR1*, with unique clonal expansion of a cluster of CD8^+^ Trm cells in UC patients compared to healthy controls (68). This cluster also expressed transcripts encoding cytotoxic granules, metabolic regulators, and the transcription factors *ZEB2* and *EOMES*. The authors then used two pre-clinical models of colitis to demonstrate a pathogenic role for CD8^+^ T cells. First, *Il10* knockout mice fed piroxicam had a marked accumulation of CD8^+^ T cells in the colon. The administration of an anti-CD8 mAb in these mice reduced weight loss and colon pathology. Secondly, the adoptive transfer of CD8^+^ T cells transduced with *Eomes* retroviral constructs into Rag1 deficient mice significantly increased weight loss and more colonic inflammation after DSS administration (68).

### B cells/plasma cells

Plasma cells are terminally differentiated B cells that secrete immunoglobulin (Ig), including IgM, IgE, IgA, and IgG. While IgA levels are highly abundant in the intestinal tissue and contribute to intestinal homeostasis (89), anti-microbial IgG in the gut is also observed at steady-state (90, 91). The large presence of anti-microbial IgG observed in IBD has renewed interest due to an *FCGR2A* variant associated with disease pathogenesis (92, 93). Thus, the role of IgG in the inflamed mucosa of IBD patients is an area of active investigation.

Huang et al. identified increases in CD138^+^ plasma cells in pediatric CD subjects by scRNA-seq (64). More specifically, seven B cell clusters including tissue resident B cells (CD44, CD69), CD19^+^ B cells, CD27^high^ B cells, CD27^low^ B cells, and two CD138^+^ plasma cell clusters expressing *IGHG1* and *IGHA1* respectively were identified. Interestingly, the tissue resident marker (CD103) was increased in plasmablasts. In addition, they observed the transition from IgA1 to IgG1 in the IBD subjects, in line with the study showing the impact of CXCR4^+^IgG^+^ plasma cells to the IBD pathogenesis (94). In another study scRNA-seq study by Boland et al. characterized different subsets of B cells and plasma cells (68). They observed an increase in the IgG1^+^ plasmablasts in UC whereas IgA2^+^ plasma cells were elevated in the healthy controls. All together, these scRNA-seq studies highlight the presence of B cells in the inflamed mucosa and bias towards IgG class-switching in plasma cells in human IBD. Uzzan and colleagues identified twenty clusters of B cells isolated from the LP when comparing healthy and UC patients (95). These clusters included naïve B cells (*IGHD*, *FCER2*, and *CD72*), memory B cells (*CD27* and *TNFRSF13B*), atypical memory B cells (*FCRL5*, *FCRL4*, and *DUSP4*), germinal center-like B cells (*AICDA*, *BCL6*, and *FAS*) and plasma cell populations based on the expression of immunoglobulin genes. Additionally, they detected an *IFNG* signature specific to a naive B cell cluster expressing *IGHD*, *FCER2*, and *CD72* suggesting a shift in the systemic humoral response towards a pro-inflammatory IgG B cell phenotype in UC.

Kong et al. collected tissue from CD patients and non-IBD donors from inflamed and non-inflamed regions of the terminal ileum and the colon and characterized the cellular networks by scRNA-seq (45). B cells were sub-clustered into plasma cells (*SDC1, MZB1, SSR4, XBP1*), B cells (*BANK, MS4A1/CD30, ASDAM28, VPREB3*) and germinal center B cells (*LRMP, GPT2, PAG1*). Plasma cells were a higher percentage of the sample in inflamed CD in the terminal ileum (45). Martin et al. described the GIMATS module, consisting of the presence of IgG-expressing plasma cells, inflammatory mononuclear phagocytes, activated T cells, and stromal cells, is expressed in a subset of ileal CD and is linked to anti-TNF therapy resistance (53).

On the other hand, in the mouse scRNA-seq study Ho et al. described decrease in the IgA producing plasma cell population at most severe time points of intestinal inflammation, whereas IgA plasma cell numbers recovered during the resolution phase (29). While differences in B cell biology have been identified between healthy and human IBD, the role of B cells has begun to be explored in pre-clinical models of IBD. In a separate study by Frede et al, they performed another longitudinal DSS-colitis study where scRNA-seq was performed on enriched B cells from the colonic LP on days 0 and 14 after DSS exposure in mice (26). While no differences in B cell cluster numbers were observed, the authors identified an expansion in an IFN-induced B cell cluster marked by *Serpina3g*, *Serpina3f*, *Stat1*, *Tgtp2*, and *Zpb1* during the mucosal healing stage of disease at day 14. Subsequent experiments where B cells were depleted by administering CD19cre-iDTR^HET^ mice diphtheria toxin during DSS administration enhanced tissue regeneration, suggesting a pathogenic role of B cells in mucosal healing (26).

## Conclusions and future perspectives

**Figure 1:**
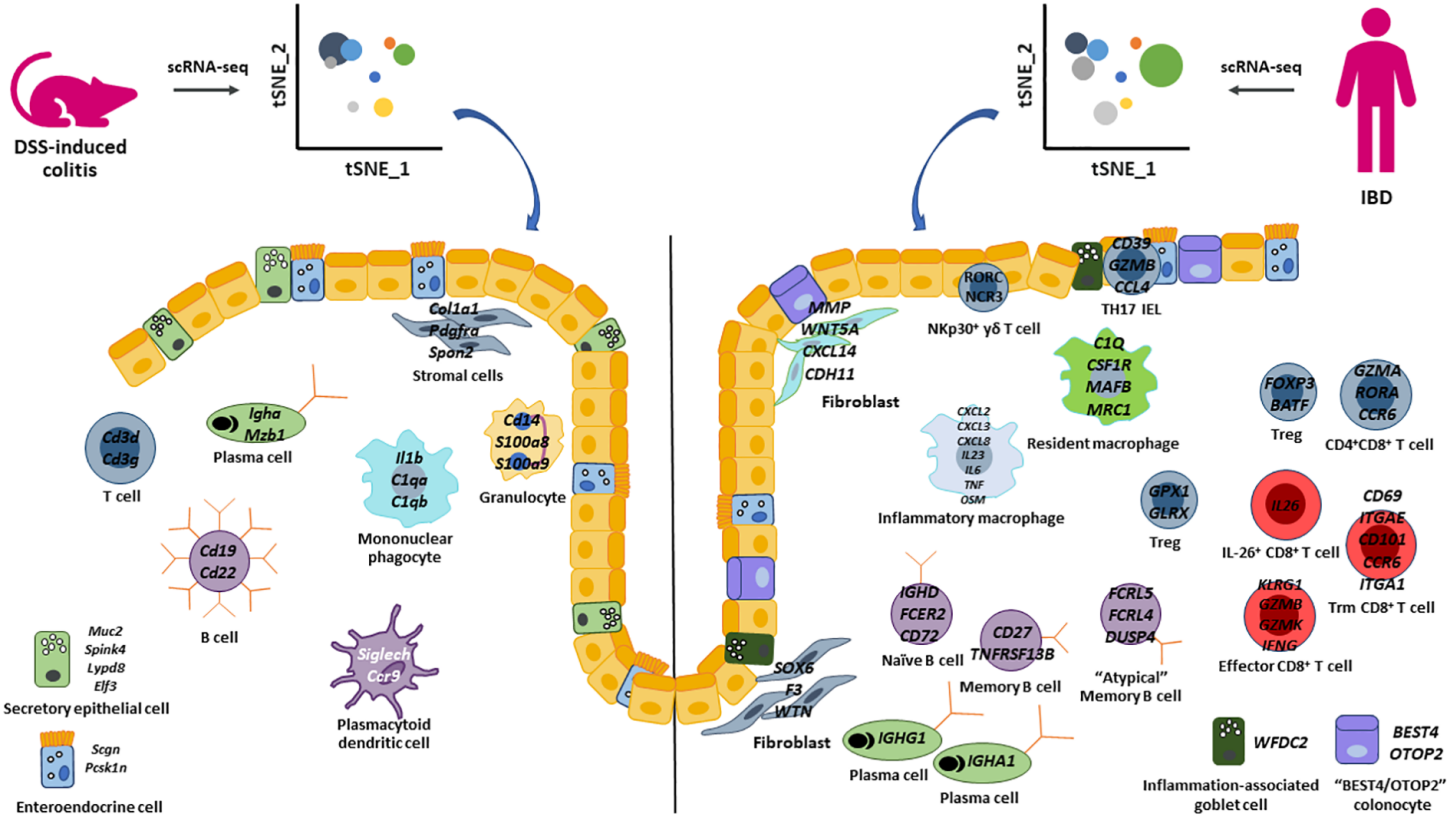
Summary of epithelial cell, stromal cell, and immune cell populations identified by scRNA-seq in the DSS-induced colitis model and human IBD. Highlighted key cell populations identified in this review that describe similar and different cell populations between the DSS-colitis pre-clinical model and human IBD.

The advances and application of single cell transcriptomics to human IBD samples have revolutionized our understanding of the complex and dynamic interactions between epithelial cell, stromal cell, and immune cell populations. Additionally, this technology has also been instrumental in identifying novel cell types and rare populations of cells, which were likely not detected previously. While numerous human IBD scRNA-seq studies were highlighted in this review, it is evident that the application of this powerful technology has not been applied enough to pre-clinical models of IBD to translate findings found in human disease. To our knowledge, the only pre-clinical model that applied scRNA-seq to generate a comprehensive landscape of epithelial cells, stromal cells, and immune cells was the longitudinal DSS study by Ho et al. (29). However, this study did not identify all cell types that have been identified by scRNA-seq in human studies (**Figure 1**). For example, a recent study combining scRNA-seq with spatial analysis (65) uncovered notable diversity within neutrophils in IBD colonic mucosa. Neutrophils were categorized into three distinct states (N1, N2, and N3) based on their unique gene expression profiles. Furthermore, novel clusters of innate lymphoid cells have been identified and contribute to intestinal inflammation (96, 97). Thus, by expanding the use of scRNA-seq to other pre-clinical models of IBD, we will be better equipped to select the most appropriate model to translate and functionally validate findings from human IBD studies.

**Figure 2:**
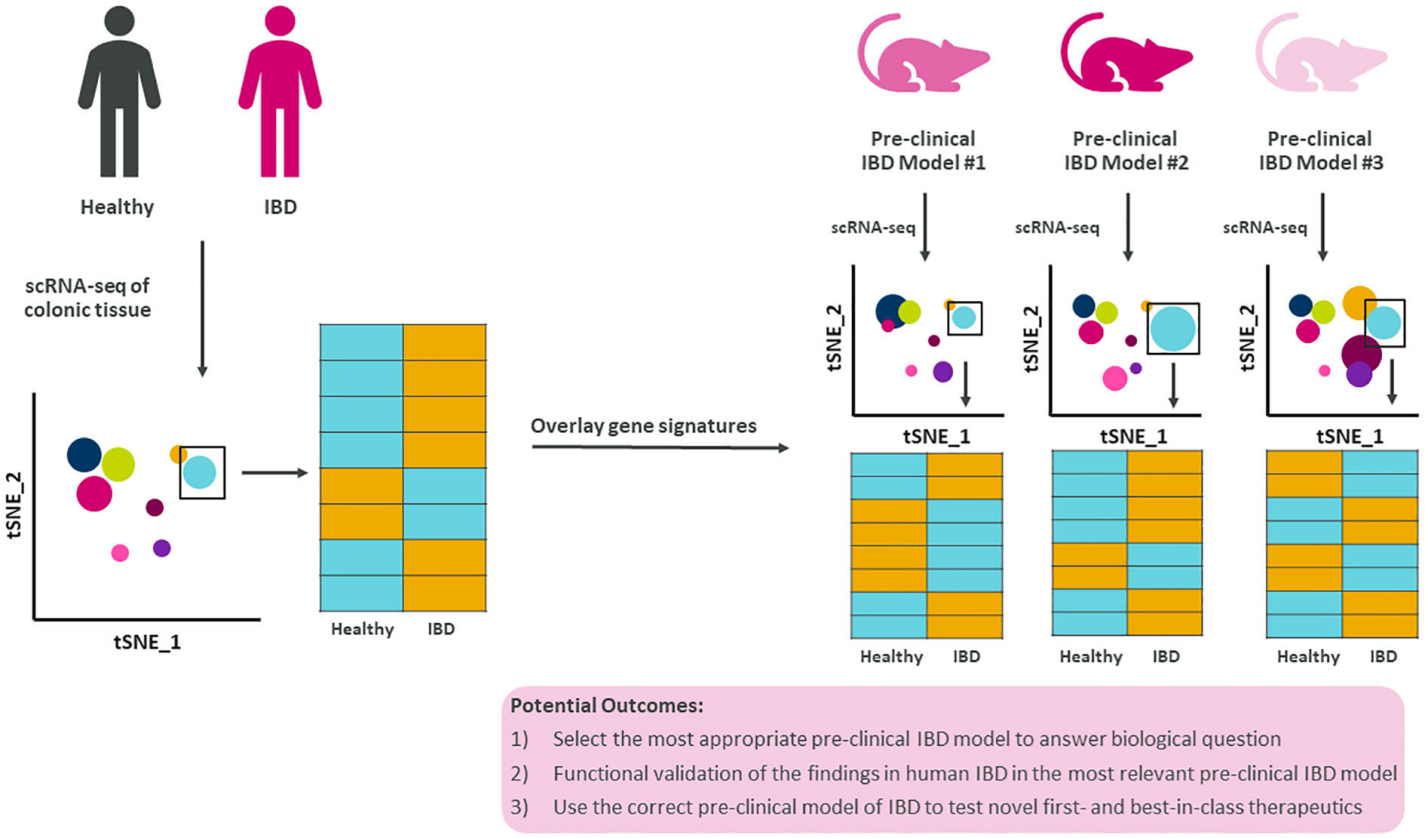
Summary figure of how translating single cell findings in human to pre-clinical models improves our understanding of disease. Human IBD gene signatures at the single cell level could be overlayed with single cell gene signatures from multiple pre-clinical models of IBD allowing for data-driven selection of the most appropriate pre-clinical model to test novel biology, questions, and therapeutics.

Furthermore, by expanding the use of scRNA-seq to all pre-clinical models to generate comprehensive disease atlases with epithelial cells, stromal cells, and immune cells will warrant the opportunity to generate novel hypotheses for therapeutic strategies (**Figure 2**). Overlaying gene signatures at the single cell level from human IBD and numerous pre-clinical models would significantly increase the likelihood of selecting the most appropriate pre-clinical model to test novel first- and best-in-class therapeutics and modalities to treat and ultimately cure IBD.

## Author contributions

EPK: Conceptualization, Writing – original draft, Writing – review & editing. AM: Conceptualization, Writing – original draft, Writing – review & editing. IK: Conceptualization, Writing – original draft, Writing – review & editing. B-EJ: Conceptualization, Writing – original draft, Writing – review & editing. RLU: Conceptualization, Writing – original draft, Writing – review & editing. KJB: Conceptualization, Supervision, Writing – original draft, Writing – review & editing. DC: Conceptualization, Supervision, Writing – original draft, Writing – review & editing. TO: Conceptualization, Supervision, Writing – original draft, Writing – review & editing.
